# Increased HDAC Activity and c-MYC Expression Mediate Acquired Resistance to WEE1 Inhibition in Acute Leukemia

**DOI:** 10.3389/fonc.2020.00296

**Published:** 2020-03-05

**Authors:** Tamara B. Garcia, Rizvan C. Uluisik, Annemie A. van Linden, Kenneth L. Jones, Sujatha Venkataraman, Rajeev Vibhakar, Christopher C. Porter

**Affiliations:** ^1^Medical Scientist Training Program, University of Colorado School of Medicine, Aurora, CO, United States; ^2^Department of Pediatrics, Emory University School of Medicine, Atlanta, GA, United States; ^3^Department of Pediatrics, University of Colorado School of Medicine, Aurora, CO, United States

**Keywords:** WEE1, c-MYC, histone deacetylase, AZD1775, adavosertib, KDM5A

## Abstract

WEE1 is a cell cycle and DNA damage response kinase that is emerging as a therapeutic target for cancer. AZD1775 is a small molecule inhibitor of WEE1, currently in early phase clinical trials as a single agent and in combination with more conventional anti-neoplastic agents. As resistance to kinase inhibitors is frequent, we sought to identify mechanisms of resistance to WEE1 inhibition in acute leukemia. We found that AZD1775 resistant cell lines are dependent upon increased HDAC activity for their survival, in part due to increased KDM5A activity. In addition, gene expression analyses demonstrate HDAC dependent increase in *MYC* expression and c-MYC activity in AZD1775 treated resistant cells. Overexpression of c-MYC confers resistance to AZD1775 in cell lines with low baseline expression. Pharmacologic inhibition of BRD4, and thereby c-MYC, partially abrogated resistance to AZD1775. Thus, acquired resistance to WEE1 inhibition may be reversed by HDAC or BRD4 inhibition in leukemia cells.

## Introduction

Inhibition of WEE1 is emerging as a promising therapeutic strategy in cancer. Many preclinical studies have demonstrated that inhibition of WEE1 enhances the efficacy of DNA damaging agents. For example, AZD1775, a small-molecule inhibitor of WEE1, synergizes with cytarabine in AML and T-ALL cells (1–3), with cisplatin in medulloblastoma cells (4), and with doxorubicin in colon adenocarcinoma cells (5). AZD1775 has also been identified as an effective monotherapy in cancers including NSCLC, ovarian clear cell carcinoma, and melanoma (6). ClinicalTrials.gov (accessed October, 2019), lists over 50 clinical studies investigating the effectiveness of AZD1775 in monotherapy and in combination with DNA damaging agents in both solid tumors and hematologic malignancies.

Despite promising preclinical results, development of resistance is a major hurdle in the clinical application of kinase inhibitors (7). Thus, the aim of the present study is to identify potential mechanisms of acquired resistance to AZD1775. Cancer cells can develop resistance to drugs by a variety of mechanisms including increased drug efflux, acquisition of mutations that prevent the binding of drug to target, and activation of compensatory survival pathways (8). Beyond these mechanisms, recent studies have described a reversible “drug-tolerant” state mediated by altered epigenetic regulation of gene expression (8–10). Specifically, increased activity of KDM5A leading to a reduction in histone 3 lysine 4 trimethylation (H3K4me3) has been shown to mediate resistance to gefitinib and cisplatin in NSCLC cells and to temozolomide in glioblastoma cells. Knockdown of KDM5A or inhibition of HDACs which bind to and regulate the activity of KDM5A was capable of reversing drug resistance in these studies (8, 9). Consistent with these studies, we demonstrate that acquired resistance to AZD1775 in three acute leukemia cell lines could be reversed with HDAC or KDM5 inhibition. Furthermore, AZD1775-resistant cells have increased c-MYC expression which is reduced upon treatment with HDAC inhibitors. Treatment with the bromodomain protein inhibitor, JQ1 (11), which inhibits c-MYC expression (12, 13), partially reversed resistance to AZD1775. Collectively, these studies indicate HDAC-mediated overexpression of c-MYC drives resistance to AZD1775 in acute leukemia cells.

## Materials and Methods

### Chemotherapies, Antibodies, and Reagents

AZD1775 was provided by AstraZeneca (Wilmington, DE). The chemical structure of AZD1775 has previously been described (14). Cytarabine and vincristine were purchased from Sigma-Aldrich (St. Louis, MO) and diluted in water. Vorinostat, panobinostat, and CPI-455 were purchased from Selleckchem (Houston, TX). TP-0906 was purchased from MedChemExpress (Monmouth Junction, NJ). JQ1 was a kind gift from the laboratory of Dr. Jay Bradner. Antibodies specific to actin, c-MYC, pCDK1 Tyr15, CDK1, pCHK1 Ser345, PARP, γH2AX, histone 3 were purchased from Cell Signaling Technology (Danvers, MA). The antibody against KDM5A was purchased from Bethyl Laboratories (Montgomery, TX), and the antibody against trimethylated histone 3 lysine 4 was purchased from Active Motif (Carlsbad, CA). Primers to detect levels of *AXL* expression relative to *GAPDH* were 5′- CAGCGCAGCCTGCATGT-3′ (Forward) and 5′- TTGGCGTTATGGGCTTCG-3′ (Reverse).

### Cell Culture and Viability

Jurkat, Molm13, and REH cells were generous gifts from the laboratories of Drs. James DeGregori and Douglas Graham. Cell lines were DNA fingerprinted by multiplex PCR using the Profiler Plus or Identifier Kits (ABI) as previously described (15), and periodically tested for Mycoplasma by PCR. Cells were cultured in RPMI with 10% FBS and penicillin/streptomycin at 37°C in humidified air supplemented with 5% CO_2_ and typically maintained in culture for no longer than 2 months at a time. However, AZD1775-resistant cells were generated by culturing Molm13, Jurkat, and REH cells in gradually increasing concentrations of 50–1,000 nM AZD1775 over 3 months. In some experiments, CellTiter-Glo Luminescent assay (Promega) was used to determine the cell viability with or without drug treatment. Around 3,000 cells per well were seeded in 100 μl media using 96 well plate. Cells were incubated 24 h prior to drug treatments. Then, the luminescent assay was applied following 72 h incubation.

### Flow Cytometry

Guava EasyCytePlus (Millipore, Billerica, MA) was also used to determine cell viability by measuring cell counts with propidium iodide exclusion. Apoptosis was assessed using Guava Nexin reagent according to the manufacturer's protocol (Millipore). Cell cycle analysis was performed using Guava Cell Cycle Reagent according to the manufacture's protocol (Millipore). A Cytoflex (Beckman Coulter) was used to serially measure GFP expression.

### Viral Transduction

The c-MYC gene was cloned into the retroviral empty vector, MSCV-IRES-GFP (MIG), to make MIG-c-MYC. Virus-containing media was prepared as previously described, with modification (16). Briefly, HEK293T cells were transfected with each plasmid of interest along with pCL-Ampho using FuGENE 6 Transfection Reagent (Promega). After a 48–72 h incubation, viral media was collected and spun for 5 min at 1,500 rpm to remove cellular debris. Jurkat, Molm-13 and REH cells were infected with viruses including MIG or MIG-c-MYC using RetroNectin protocol as instructed by the manufacturer (Clontech). Then cells incubated for 24 h at 37°C and GFP expression was determined with flow cytometry.

### RNA-Seq

AZD1775-sensitive and -resistant Jurkat cells were treated with panobinostat (10 nM) and/or AZD1775 (1 μM) for 24 h. Total RNA was extracted using a RNeasy kit (Qiagen Inc., Valencia, CA). cDNA libraries were constructed for each sample using the TruSeq Stranded RNA kit (Illumina Inc., San Diego, CA) according to the manufacturer's protocol. The unique cDNA libraries were sequenced as single-pass 50 bp reads on the Illumina HiSeq4000 platform at the University of Colorado Genomics and Sequencing Core Facility. The resulting sequences were analyzed using a custom pipeline consisting of gSNAP, Cufflinks, and R for sequence alignment and identification of differential gene expression as previously described (17, 18). Genes with a false-discovery rate (FDR) <0.05 were analyzed using Ingenuity Pathways Analysis (Qiagen, Germantown, MD) to identify pathways modified in sensitive and resistant cells treated with AZD1775 and/or panobinostat.

### Statistical Analysis

Data analysis and graphing was performed using GraphPad Prism 5 (GraphPad Software, La Jolla, CA). Unless otherwise indicated, graphs represent the mean from a minimum of three biological replicate experiments, and error bars portray the standard error of the mean. One-way ANOVA was used to compare three or more samples with a single variable. Two-way ANOVA was used to compare three or more samples with two variables, with Bonferroni's post-test analysis. Non-linear regression was used to generate dose-response curves and determine IC50 values. Two-way repeated measures ANOVA with Tukey's correction for multiple comparison was used to compare the percentage of GFP^+^ cells over time.

## Results

### Generation and Characterization of AZD1775-Resistant Acute Leukemia Cell Lines

**Figure 1:**
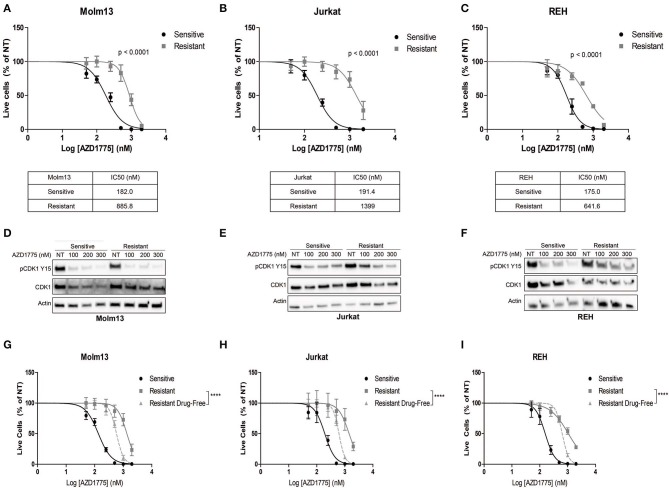
Generation and characterization of AZD1775-resistant cell lines. AZD1775 resistance was generated in Molm13, Jurkat and REH cell lines by culturing these cell lines in media containing 50–10,000 nM AZD1775 over a period of 3 months. **(A–C)** AZD1775-naive and -resistant Molm13 **(A)**, Jurkat **(B)**, and REH **(C)** cells were treated with 50–2,000 nM AZD1775 for 72 h. Cellular proliferation rates and viability was determined by flow cytometry. Viable cell numbers normalized to cells receiving no treatment (NT) are shown. Results are shown as mean ± SEM from three independent experiments. IC50 values for each cell line are displayed below the corresponding graph. **(D–F)** Molm13 **(D)**, Jurkat **(E)**, and REH **(F)** sensitive and resistant cells were treated with the indicated concentrations of AZD1775 for 24 h after which protein lysates were subjected to western blotting with antibodies specific to pCDK1 tyrosine 15, CDK1, and actin. **(G–I)** Molm13 **(G)**, Jurkat **(H)**, and REH **(I)** cells resistant to AZD1775 were cultured in drug-free media for 40 passages. Sensitive, resistant cells and resistant cells cultured in drug-free media were treated with 50–2,000 nM AZD1775 for 72 h. Viable cell counts normalized to cells receiving no treatment are shown. Results are shown as mean ± SEM from three independent experiments. ^****^P < 0.0001.

We generated resistance to AZD1775 in three acute leukemia cell lines with diverse phenotypes and genetic backgrounds (19). Each resistant cell line displayed significantly less sensitivity to the anti-proliferative effects of AZD1775 (Figures 1A–C). Western blots demonstrated that WEE1 expression levels were similar in parental and resistant cell lines (Supplementary Figure 1). Reverse transcription of WEE1 mRNA, amplification of overlapping regions of the entire gene by PCR, and Sanger sequencing from AZD1775-resistant cells identified no mutations at any point in the *WEE1* gene (data not shown). Additionally, resistant cells treated with AZD1775 at concentrations lower than the IC50 display reduced WEE1 activity as evidenced by decreased phosphorylation of CDK1 at tyrosine 15 (Figures 1D–F). Thus, resistance to AZD1775 is not due to reduced activity of the drug in any of the three resistant cell lines, excluding altered expression, gatekeeper mutations and enhanced drug efflux as mechanisms of resistance to AZD1775 in these cell lines (7, 20). Recently, increased expression and activation of AXL was demonstrated to mediate primary and acquired resistance to AZD1775 in small-cell lung cancer (21). In contrast to those findings, we did not observe consistent increases in AXL expression or sensitivity to AXL inhibition in resistant cell lines, as compared to AZD1775 naive cells, nor consistent combinatorial activity of AXL and WEE1 inhibition in AZD1775 resistant cells (Supplementary Figure 1), suggesting that alternative mechanisms of resistance to AZD1775 exist.

Drug resistance due to altered cellular signaling or gene expression in the absence of mutations can be reversible when resistant cells are released from the selective pressure of the drug (8, 9). To determine whether resistance to AZD1775 is reversible, each resistant cell line was cultured in drug-free media for 40 passages. Resistant cells cultured in drug-free media re-acquire sensitivity to AZD1775 compared with resistant cells maintained in AZD1775 (Figures 1G–I). While the resistant cells cultured in drug-free media are still less sensitive to AZD1775 compared to the AZD1775-naive cell lines, these data indicate resistance to AZD1775 is at least partially reversible upon drug removal.

Treatment with AZD1775 ultimately results in DNA damage (22). Thus, prevention of DNA damage or enhanced DNA damage repair might induce resistance to WEE1 inhibition, as well as additional DNA damaging agents. To assess this, sensitive and resistant cells were treated with cytarabine (Ara-C) and vincristine, two conventional chemotherapy agents used to treat leukemia. AZD1775-resistant Molm13 and REH cells were equally sensitive to Ara-C compared with AZD1775-naive cells (Supplementary Figures 2A,C). Resistant Jurkat cells were slightly more sensitive to Ara-C than the sensitive cells (Supplementary Figure 2B). Resistance to AZD1775 was not associated with reduced sensitivity to vincristine in Jurkat and REH cells, but AZD1775-resistant Molm13 cells were less sensitive to vincristine [IC50: 0.336 nM (sensitive cells) vs. 0.613 nM (resistant cells)] (Supplementary Figures 2D–F). The variable response to conventional chemotherapeutics suggests these cell lines may vary in ability to repair DNA damage. However, cells resistant to AZD1775 are not broadly resistant to conventional DNA damaging agents.

While some studies suggest AZD1775 might be effective in single-agent therapy, the majority of pre-clinical studies with AZD1775 have been performed in combination with DNA damaging agents (6). Thus, we sought to determine whether cells resistant to single-agent AZD1775 were also resistant to AZD1775 combined with Ara-C. Consistent with previous results from our lab (1, 2, 15), AZD1775 enhanced the anti-proliferative effects of Ara-C in AZD1775-naive cell lines. However, AZD1775 did not sensitize resistant cells to Ara-C at concentrations effective in sensitive cells (Supplementary Figure 3). Therefore, cells resistant to single-agent AZD1775 treatment also display reduced sensitivity to AZD1775 combined with Ara-C.

### HDAC Inhibition Increases Sensitivity to AZD1775 in Resistant Cell Lines

**Figure 2:**
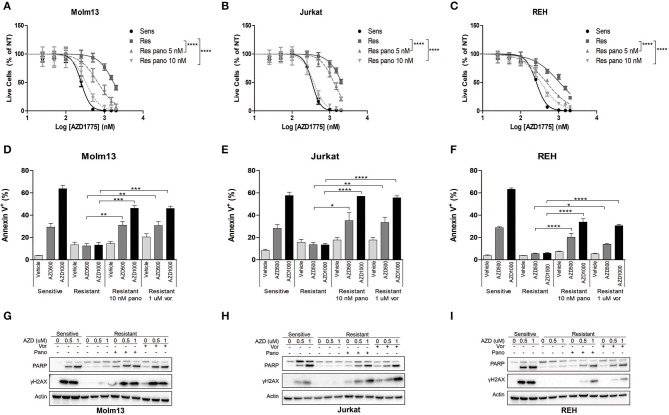
HDAC inhibition sensitizes resistant cells to AZD1775. **(A–C)** AZD1775-naive and -resistant Molm13 **(A)**, Jurkat **(B)**, and REH **(C)** cells were treated with the indicated concentrations of panobinostat and/or 25–2,000 nM AZD1775 for 72 h. Viable cell counts normalized to cells receiving no AZD1775 treatment (NT) are shown. Results are shown as mean ± SEM from a minimum of three independent experiments. ****P < 0.0001. **(D–F)** AZD1775-naive and -resistant Molm13 **(D)**, Jurkat **(E)**, and REH **(F)** cells were treated with panobinostat (10 nM), vorinostat (1 μM), and/or AZD1775 (500 or 1,000 nM) for 24 h and stained with Annexin V-PE. Results are displayed as mean ± SEM of three independent experiments. *P < 0.05, **P < 0.01, ***P < 0.001, ****P < 0.0001. **(G–I)** AZD1775-naive and -resistant Molm13 **(G)**, Jurkat **(H)**, and REH **(I)** cells were treated with panobinostat (10 nM), vorinostat (1 μM), and/or AZD1775 (500 or 1,000 nM) for 24 h after which protein lysates were subjected to western blotting with antibodies specific to PARP, γH2AX, and actin.

The observation that resistance to AZD1775 is partially reversible after prolonged treatment in drug-free media suggested that an altered epigenetic landscape could promote resistance to AZD1775. HDAC inhibition has been demonstrated to abrogate resistance to a number of drugs including imatinib in CML cells and gefitinib and cisplatin in lung cancer cells (10). Consistent with these findings, treatment with pan-HDAC inhibitors panobinostat or vorinostat enhanced the anti-proliferative effects of AZD1775 in resistant Molm13, Jurkat, and REH cells (Figures 2A–C, Supplementary Figure 4).

Next, cells were treated with panobinostat, vorinostat, and/or AZD1775 to determine whether HDAC inhibition could restore DNA damage and apoptosis induction in response to WEE1 inhibition. Naive cells treated with AZD1775 display a dose-dependent increase in Annexin V positive cells indicating an increase in apoptosis (Figures 2D–F). None of the three resistant cell lines have increased apoptosis induction over baseline upon treatment with AZD1775 (Figures 2D–F). HDAC inhibition alone does not increase apoptosis in resistant cells; however, it does promote a dose-dependent increase in apoptosis in response to AZD1775 as indicated by increased Annexin V positive cells. Further supporting an increase in apoptosis, treatment with panobinostat or vorinostat resulted in an increase in cleaved PARP in resistant cells treated with AZD1775 (Figures 2G–I). HDAC inhibition also increases DNA damage induction, as evidenced by elevated γH2AX, in resistant cells treated with AZD1775 (Figures 2G–I).

We also questioned whether altered cell cycle progression due to HDAC inhibition (23) could contribute to the re-sensitization to AZD1775. Consistent with previous reports (24), inhibition of WEE1 leads to arrest in S phase in all three AZD1775-naive cell lines as well as an increase in cells in G2/M phase in Jurkat and REH AZD1775-naive cells (Supplementary Figures 5A–C), but not in any of the AZD1775-resistant cells. Treatment of resistant cells with HDAC inhibitors produces a slight increase in S and/or G2/M phase cells (Supplementary Figures 5A–C). However, this cell cycle arrest is less pronounced than that in naive cells treated with AZD1775. Thus, while altered cell cycle progression might contribute to the increased sensitivity to AZD1775, it does not appear to be the primary mechanism by which HDAC inhibitors restore sensitivity to AZD1775 in resistant cells.

HDAC inhibitors including vorinostat have been reported to synergize with AZD1775 in AML cells without acquired resistance to WEE1 inhibition (25), which we were able to corroborate (Supplementary Figure 6). In addition, AZD1775 treatment was reported to lead to increased phosphorylation of CHK1, and HDAC inhibition abrogated CHK1 phosphorylation leading to decreased DNA damage repair and subsequent apoptosis (25). Thus, we sought to determine whether resistance to AZD1775 was the result of increased CHK1 activity promoting DNA damage repair. AZD1775 treatment promoted increased phosphorylation of CHK1 in AZD1775-naive Jurkat cells (Supplementary Figure 5D). AZD1775-resistant Jurkat cells do not have increased CHK1 phosphorylation with AZD1775, and this is not increased with HDAC inhibition (Supplementary Figure 5D). Thus, increased DNA damage repair mediated by ATR/CHK1 is not responsible for resistance to AZD1775 in Jurkat cells.

### Inhibition of KDM5A Enhances the Anti-proliferative Effects of AZD1775 in Resistant Cells

**Figure 3:**
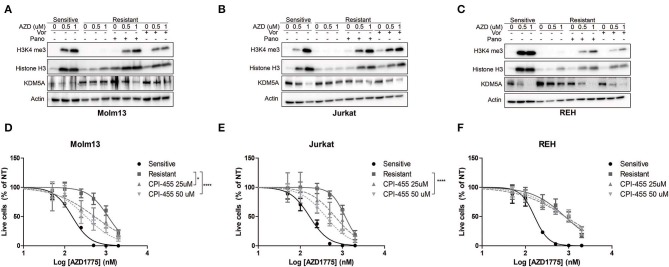
Altered H3K4 trimethylation contributes to AZD1775 resistance. **(A–C)** AZD1775-naive and -resistant Molm13 **(A)**, Jurkat **(B)**, and REH **(C)** cells were treated with panobinostat (10 nM), vorinostat (1 μM), and/or AZD1775 (0.5 or 1 μM) for 24 h after which protein lysates were subjected to western blotting with antibodies specific to H3K4me3, histone H3, KDM5A, and actin. **(D–F)** AZD1775-naive and -resistant Molm13 **(D)**, Jurkat **(E)**, and REH **(F)** cells were treated with the indicated concentrations of CPI-455 and/or 50–2,000 nM AZD1775 for 72 h. Viable cell counts are normalized to cells receiving no AZD1775 treatment (NT). Results are displayed as mean ± SEM from three independent experiments. *P < 0.05, ****P > 0.0001.

Previous reports have demonstrated that inhibition of HDACs abrogates drug resistance by reducing the activity of KDM5A, an enzyme that removes di- and tri-methyl groups from histone 3 lysine 4 (8, 9). Therefore, we questioned whether HDAC inhibition altered H3K4me3 in resistant cells treated with AZD1775. AZD1775 leads to increased H3K4me3 in sensitive cells but not in resistant cells (Figures 3A–C). The increase in H3K4me3 in sensitive cells is accompanied by decreased levels of KDM5A while KDM5A remains elevated in resistant cells treated with AZD1775. The increase in H3K4me3 and decrease in KDM5A in response to WEE1 inhibition are restored in resistant cells treated with panobinostat or vorinostat (Figures 3A–C). Notably, upon WEE1 inhibition, we observed increased levels of histone H3 in sensitive, and to a lesser extent, resistant cell lines, consistent with a role for WEE1 in regulating histone expression levels (26). Nonetheless, treatment with an inhibitor of KDM5 isoforms, CPI-455, enhanced the anti-proliferative effects of AZD1775 in resistant Molm13 and Jurkat cells (Figures 3D,E). CPI-455 treatment did not sensitize resistant REH cells to AZD1775 (Figure 3F). Thus, in combination with AZD1775, HDAC inhibition reduces KDM5A protein levels and increases H3K4me3 in all resistant cell lines, and inhibition of KDM5 enhances sensitivity to AZD1775 in two of three resistant lines. This suggests altered histone methylation contributes to AZD1775 resistance, at least in Molm13 and Jurkat cells, and also highlights a difference in the mechanisms of AZD1775 resistance in REH cells.

### c-MYC Expression Is Regulated by HDACs and Contributes to AZD1775 Resistance

**Figure 4:**
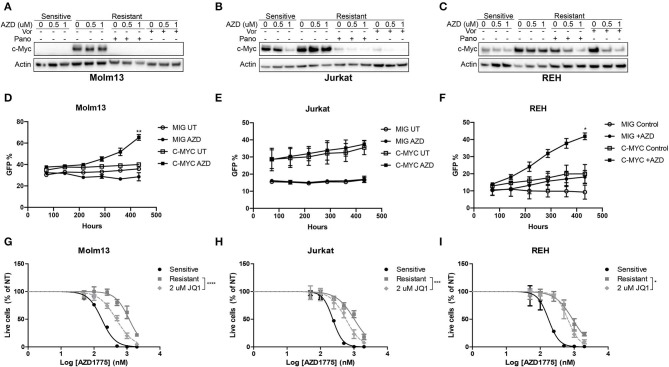
c-MYC expression contributes to AZD1775 resistance. **(A–C)** AZD1775-naive and -resistant Molm13 **(A)**, Jurkat **(B)**, and REH **(C)** cells were treated with panobinostat (10 nM), vorinostat (1 μM), and/or AZD1775 (0.5 or 1 μM) for 24 h after which protein lysates were subjected to western blotting with antibodies specific to c-MYC and actin. **(D–F)** AZD1775-naïve Molm13 **(D)**, Jurkat **(E)** and REH **(F)** cells were transfected with MIG or MIG-c-MYC plasmids and cultured with 200 nM AZD1775 or 0.02% DMSO (UT). The percentage of GFP^+^ cells was measured and the cells were re-plated with fresh media and drug every 72 h. **(G–I)** AZD1775-naive and -resistant Molm13 **(G)**, Jurkat **(H)**, and REH **(I)** cells were treated with the indicated concentration of JQ1 and/or 50–2,000 nM AZD1775 for 72 h. Viable cell counts normalized to cells receiving no AZD1775 treatment (NT) are shown. Results are displayed as mean ± SEM from three independent experiments. *P < 0.05, ** *P* < 0.01, ***P < 0.001, ****P < 0.0001.

Next, we sought to identify changes in gene expression between AZD1775-naive and -resistant cells treated with AZD1775 and/or panobinostat. To accomplish this, AZD1775-naive and -resistant Jurkat cells were treated with panobinstat and/or AZD1775 for 24 h, and extracted RNA was subjected to RNAseq. Ingenuity Pathway Analysis identified increased expression and activity of c-MYC in resistant cells treated with AZD1775 compared with sensitive cells treated with AZD1775 (Supplementary Figure 7A). Addition of panobinostat to resistant cells treated with AZD1775 resulted in decreased c-MYC expression as well as altered expression of some c-MYC target genes (Supplementary Figure 7B). We confirmed that elevated c-MYC protein levels were observed in all three resistant cell lines treated with AZD1775 compared with sensitive cells, and this was reduced by addition of panobinostat or vorinostat (Figures 4A–C). To directly test whether c-MYC can confer resistance to WEE1 inhibition, we overexpressed c-MYC in AZD1775 sensitive cell lines. Jurkat, Molm13 and REH cell lines were transfected with MIG or MIG-c-MYC, and the percentage of GFP^+^ cells was monitored over time. As expected, c-MYC expression provided a proliferative advantage over time in each of the cell lines to varying degrees in the absence of drug treatment. However, in the presence of AZD1775, a significant proliferation advantage was observed in REH and Molm13 cells which over expressed c-MYC (Figures 4D–F). Notably, no such advantage was seen in Jurkat cells, which express high levels of c-Myc at baseline (Figure 4B).

To determine whether inhibition of c-MYC activity could be contributing to resistance to AZD1775, each resistant cell line was treated with JQ1, a small-molecule inhibitor of the bromodomain and extraterminal domain (BET) family proteins that has been shown to reduce c-MYC expression and inhibit MYC-dependent transcription by reducing activity of BET family member BRD4 (12, 13). Consistent with a role for c-MYC in mediating resistance to AZD1775, treatment with JQ1 enhanced the anti-proliferative effects of AZD1775 in each resistant cell line (Figures 4G–I). Together, these results indicate that AZD1775-resistant cells have elevated c-MYC expression which can be abrogated by HDAC inhibition, and treatment with JQ1 can partially restore sensitivity to AZD1775.

## Discussion

Previous studies from our lab and others have demonstrated that the small-molecule inhibitor of WEE1, AZD1775, is a promising antineoplastic agent alone and in combination with conventional DNA damaging agents and some targeted agents (1, 2, 27). However, as with other tyrosine kinase inhibitors, resistance to AZD1775 is likely to occur. In this study, we sought to identify potential mechanisms of acquired resistance to AZD1775 at clinically relevant concentrations (28) in three genetically diverse acute leukemia cell lines. AZD1775 resistance was not the result of elevated drug efflux or a gatekeeper mutation in the *WEE1* gene. While there was some difference in sensitivity to Ara-C and vincristine between the three cell lines, resistant Molm13, Jurkat, and REH cells were not consistently resistant to these DNA damaging agents. While this data does not confirm resistance to AZD1775 is pathway specific, it does demonstrate that cells resistant to AZD1775 are not broadly drug resistant. Resistance to AZD1775 was partially reversed after prolonged culture in drug-free media, consistent with a non-mutational mechanism of resistance. This reversibility suggests patients who develop resistance to AZD1775 may benefit from re-treatment after a “drug holiday.”

Previous studies have identified a population of reversible “drug-tolerant” cells in which tolerance is mediated by an altered epigenetic landscape that can be reversed by inhibition of HDACs or KDM5A (8, 9). Consistent with these reports, we identified HDACs as mediators of acquired resistance to AZD1775 in each of our three resistant cell lines. In addition to increasing sensitivity to AZD1775, HDAC inhibition restores reduced KDM5A protein levels and increased H3K4 trimethylation in response to AZD1775 treatment in resistant cells. We did not interrogate whether AZD1775 resistance is the result of HDAC-mediated alterations in histone acetylation or of KDM5A-mediated changes in histone methylation. However, the association between HDAC inhibition and reduced KDM5A protein levels and re-sensitization to AZD1775 with either HDAC or KDM5 inhibition suggests one or both of the KDM5A-containing HDAC complexes, NuRD or SIN3B-containing complex, may mediate acquired AZD1775 resistance. Notably, KDM5 inhibition did not sensitize REH resistant cells to AZD1775. While this could indicate that HDACs mediate resistance in REH cells through mechanisms different from those in Molm13 and Jurkat cells, compensatory activity of KDM1A on H3K4me3 could also be possible in REH cells (29). The similarity of dependence on HDAC and KDM5A activity for AZD1775 resistance in acute leukemia cells, gefitinib resistance in NSCLC cells (8), and temozolomide resistance in glioblastoma cells (9), suggests multiple cancer types may adopt an altered epigenetic landscape that promotes drug tolerance in response to agents with differing mechanisms of action.

Gene expression analysis identified increased expression of c-MYC in resistant cells treated with AZD1775 which was reduced with HDAC inhibition. c-MYC binds to and regulates the transcription of up to 15% of all genes and influences many cellular processes including cell cycle progression, DNA replication, survival, and differentiation (30). Thus, altered expression of many genes may contribute to AZD1775 resistance. Notably, c-MYC regulates multiple genes involved in nucleotide synthesis (31). Inhibition of WEE1 leads to nucleotide shortage and subsequent replication fork collapse (22), so c-MYC could function to increase nucleotide production in resistant cells. Increased nucleotide pools could prevent DNA damage accumulation in the context of WEE1 inhibition, and this is consistent with the observations of reduced γH2AX and CHK1 phosphorylation in resistant cells treated with AZD1775.

In conclusion, this report describes a novel mechanism of resistance to AZD1775 in three acute leukemia cell lines mediated by increased HDAC activity leading to increased c-MYC expression and activity. Whether these pathways will be important with other drugs that inhibit WEE1 activity remains to be determined, and as clinical trials with AZD1775 progress, future work using primary patient samples will be required to determine whether patients develop resistance to AZD1775 in a mechanism similar to that observed our cell lines. However, this work provides preliminary evidence for potential therapeutic options in the setting of AZD1775 resistance.

## Data Availability Statement

The datasets generated for this study can be found in the NCBI Gene Expression Omnibus (GSE145129).

## Author Contributions

TG, RU, AL, and SV performed experiments, analyzed data, and wrote or edited the manuscript. KJ analyzed data. RV and CP directed the research, analyzed data, and edited the manuscript.

### Conflict of Interest

The authors declare that the research was conducted in the absence of any commercial or financial relationships that could be construed as a potential conflict of interest.

## References

[1] Ford JB Baturin D Burleson TM Van Linden AA Kim YM Porter CC AZD1775 sensitizes T cell acute lymphoblatic leukemia cells to cytarabine by promoting apoptosis over DNA repair. Oncotarget. (2015) 6:28001–10. 10.18632/oncotarget.4830 26334102 PMC4695040

[2] Porter CC Kim J Fosmire S Gearheart CM van Linden A Baturin D. Integrated genomic analyses identify WEE1 as a critical mediator of cell fate and a novel therapeutic target in acute myeloid leukemia. Leukemia. (2012) 26:1266–76. 10.1038/leu.2011.392 22289989 PMC3678731

[3] Tibes R Bogenberger JM Chaudhuri L Hagelstrom RT Chow D Buechel ME. RNAi screening of the kinome with cytarabine in leukemias. Blood. (2012) 119:2863–72. 10.1182/blood-2011-07-367557 22267604 PMC8221104

[4] Harris PS Venkataraman S Alimova I Birks DK Balakrishnan I Cristiano B. Integrated genomic analysis identifies the mitotic checkpoint kinase WEE1 as a novel therapeutic target in medulloblastoma. Mol Cancer. (2014) 13:72. 10.1186/1476-4598-13-72 24661910 PMC3987923

[5] Hirai H Arai T Okada M Nishibata T Kobayashi M Sakai N. MK-1775, a small molecule Wee1 inhibitor, enhances anti-tumor efficacy of various DNA-damaging agents, including 5-fluorouracil. Cancer Biol Ther. (2010) 9:514–22. 10.4161/cbt.9.7.11115 20107315

[6] Guertin AD Li J Liu Y Hurd MS Schuller AG Long B Preclinical evaluation of the WEE1 inhibitor MK-1775 as a single agent anticancer therapy. Mol Cancer Ther. (2013) 3:1442–52. 10.1158/1535-7163.MCT-13-0025 23699655

[7] Barouch-Bentov R Sauer K. Mechanisms of drug-resistance in kinases. Exp Opin Investig Drugs. (2011) 20:153–208. 10.1517/13543784.2011.546344 21235428 PMC3095104

[8] Sharma SV Lee DY Li B Quinlan MP Takahashi F Maheswaran S. A chromatin-mediated reversible drug-tolerant state in cancer cell subpopulations. Cell. (2010) 141:69–80. 10.1016/j.cell.2010.02.027 20371346 PMC2851638

[9] Banelli B Carra E Barbieri F Wurth R Parodi F Pattarozzi A. The histone demethylase KDM5A is a key factor for the resistance to temozolomide in glioblastoma. Cell Cycle. (2015) 14:3418–29. 10.1080/15384101.2015.1090063 26566863 PMC4825557

[10] Pribluda A de la Cruz CC Jackson EL. Intratumoral heterogeneity: from diversity comes resistance. Clin Cancer Res. (2015) 21:2916–23. 10.1158/1078-0432.CCR-14-1213 25838394

[11] Filippakopoulos P Qi J Picaud S Shen Y Smith WB Fedorov O. Selective inhibition of BET bromodomains. Nature. (2010) 468:1067–73. 10.1038/nature09504 20871596 PMC3010259

[12] Zuber J Shi J Wang E Rappaport AR Herrmann H Sison EA. RNAi screen identifies Brd4 as a therapeutic target in acute myeloid leukaemia. Nature. (2011) 478:524–8. 10.1038/nature10334 21814200 PMC3328300

[13] Venkataraman S Alimova I Balakrishnan I Harris P Birks DK Griesinger A. Inhibition of BRD4 attenuates tumor cell self-renewal and suppresses stem cell signaling in MYC driven medulloblastoma. Oncotarget. (2014) 5:2355–71. 10.18632/oncotarget.1659 24796395 PMC4058011

[14] Hirai H Iwasawa Y Okada M Arai T Nishibata T Kobayashi M. Small-molecule inhibition of Wee1 kinase by MK-1775 selectively sensitizes p53-deficient tumor cells to DNA-damaging agents. Mol Cancer Ther. (2009) 8:2992–3000. 10.1158/1535-7163.MCT-09-0463 19887545

[15] Van Linden AA Baturin D Ford JB Fosmire SP Gardner L Korch C. Inhibition of Wee1 sensitizes cancer cells to antimetabolite chemotherapeutics *in vitro* and *in vivo*, independent of p53 functionality. Mol Cancer Ther. (2013) 12:2675–84. 10.1158/1535-7163.MCT-13-0424 24121103 PMC3897395

[16] Porter CC DeGregori J. Interfering RNA-mediated purine analog resistance for *in vitro* and *in vivo* cell selection. Blood. (2008) 112:4466–74. 10.1182/blood-2008-03-146571 18587011 PMC2597122

[17] Baird NL Bowlin JL Cohrs RJ Gilden D Jones KL. Comparison of varicella-zoster virus RNA sequences in human neurons and fibroblasts. J Virol. (2014) 88:5877–80. 10.1128/JVI.00476-14 24600007 PMC4019124

[18] Wu TD Nacu S. Fast and SNP-tolerant detection of complex variants and splicing in short reads. Bioinformatics. (2010) 26:873–81. 10.1093/bioinformatics/btq057 20147302 PMC2844994

[19] Garcia TB Fosmire SP Porter CC. Increased activity of both CDK1 and CDK2 is necessary for the combinatorial activity of WEE1 inhibition and cytarabine. Leuk Res. (2018) 64:30–3. 10.1016/j.leukres.2017.11.004 29175378 PMC5929465

[20] Gottesman MM. Mechanisms of cancer drug resistance. Annu Rev Med. (2002) 53:615–27. 10.1146/annurev.med.53.082901.103929 11818492

[21] Sen T Tong P Diao L Li L Fan Y Hoff J. Targeting AXL and mTOR pathway overcomes primary and acquired resistance to WEE1 inhibition in small-cell lung cancer. Clin Cancer Res. (2017) 23:6239–53. 10.1158/1078-0432.CCR-17-1284 28698200 PMC5882197

[22] Beck H Nahse-Kumpf V Larsen MS O'Hanlon KA Patzke S Holmberg C. Cyclin-dependent kinase suppression by WEE1 kinase protects the genome through control of replication initiation and nucleotide consumption. Mol Cell Biol. (2012) 32:4226–36. 10.1128/MCB.00412-12 22907750 PMC3457333

[23] Finzer P Kuntzen C Soto U zur Hausen H Rosl F. Inhibitors of histone deacetylase arrest cell cycle and induce apoptosis in cervical carcinoma cells circumventing human papillomavirus oncogene expression. Oncogene. (2001) 20:4768–76. 10.1038/sj.onc.1204652 11521189

[24] Beck H Nahse V Larsen MS Groth P Clancy T Lees M. Regulators of cyclin-dependent kinases are crucial for maintaining genome integrity in S phase. J Cell Biol. (2010) 188:629–38. 10.1083/jcb.200905059 20194642 PMC2835936

[25] Zhou L Zhang Y Chen S Kmieciak M Leng Y Lin H. A regimen combining the Wee1 inhibitor AZD1775 with HDAC inhibitors targets human acute myeloid leukemia cells harboring various genetic mutations. Leukemia. (2015) 29:807–18. 10.1038/leu.2014.296 25283841 PMC4387110

[26] Mahajan K Fang B Koomen JM Mahajan NP. H2B Tyr37 phosphorylation suppresses expression of replication-dependent core histone genes. Nat Struct Mol Biol. (2012) 19:930–7. 10.1038/nsmb.2356 22885324 PMC4533924

[27] Garcia TB Snedeker JC Baturin D Gardner L Fosmire SP Zhou C A small molecule inhibitor of WEE1, AZD1775, synergizes with olaparib by impairing homologous recombination and enhancing DNA damage and apoptosis in acute leukemia. Mol Cancer Ther. (2017) 10:2058–68. 10.1158/1535-7163.MCT-16-0660 PMC5628125 28655785

[28] Do K Wilsker D Ji J Zlott J Freshwater T Kinders RJ Phase I study of single-agent AZD1775 (MK-1775), a Wee1 kinase inhibitor, in patients with refractory solid tumors. J Clin Oncol. (2015) 30:3409–15. 10.1200/JCO.2014.60.4009 PMC4606059 25964244

[29] Pajtler KW Weingarten C Thor T Künkele A Heukamp LC Büttner R. The KDM1A histone demethylase is a promising new target for the epigenetic therapy of medulloblastoma. Acta Neuropathol Commun. (2013) 1:19. 10.1186/2051-5960-1-19 24252778 PMC3893444

[30] Patel JH Loboda AP Showe MK Showe LC McMahon SB. Analysis of genomic targets reveals complex functions of MYC. Nat Rev Cancer. (2004) 4:562–8. 10.1038/nrc1393 15229481

[31] Liu Y-C Li F Handler J Huang CRL Xiang Y Neretti N. Global regulation of nucleotide biosynthetic genes by c-Myc. PLoS ONE. (2008) 3:e2722. 10.1371/journal.pone.0002722 18628958 PMC2444028

