# Supplementary material for: Increased HDAC Activity and c-MYC Expression Mediate Acquired Resistance to WEE1 Inhibition in Acute Leukemia
Source: Front Oncol. 2020 Mar 5;10:296. doi: 10.3389/fonc.2020.00296 (PMC7066074; doi:10.3389/fonc.2020.00296)
Supplement: Supplementary file 1 [file Data_Sheet_1.docx]

**
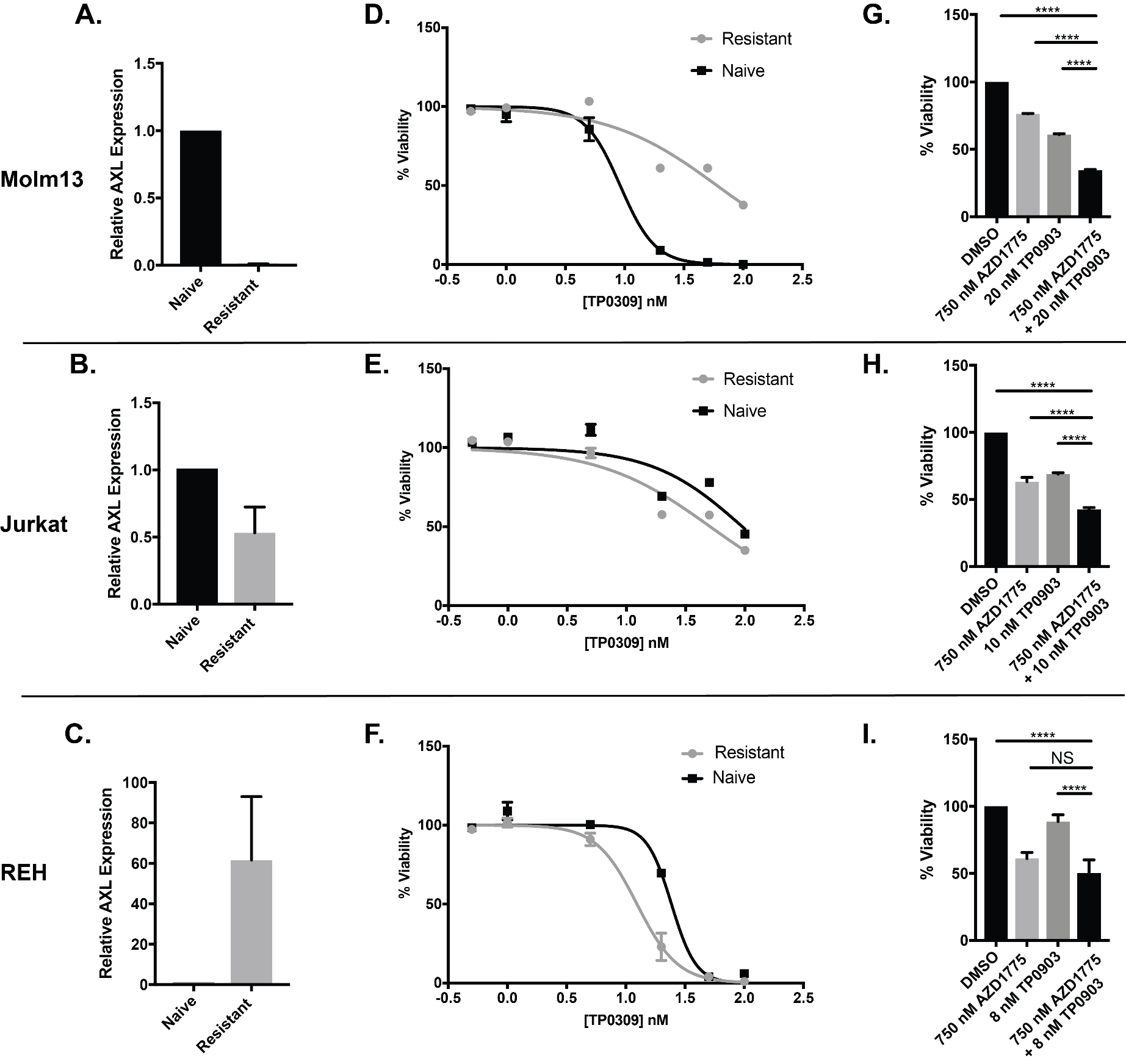
Supplementary Figure 1. Resistance to WEE1 inhibition is not consistently due to increased WEE1 expression or AXL dependency. Inset.** Western blot for WEE1 showing no change in expression levels between AZD1775 -naïve and -resistant cell lines. **A, B, C.** RNA from AZD1775-sensitive (naïve) and -resistant Molm13, Jurkat, and REH cells was reverse-transcribed and evaluated with primers and probes specific for AXL expression levels. **D, E, F.** AZD1775-sensitive and -resistant Molm13, Jurkat, and REH cells were treated with the AXL inhibitor TP-0903 at the indicated concentrations. **G, H, I.** AZD1775-resistant Molm13, Jurkat, and RE H cells were treated with AZD1775 and TP-0903 at the indicated concentrations.


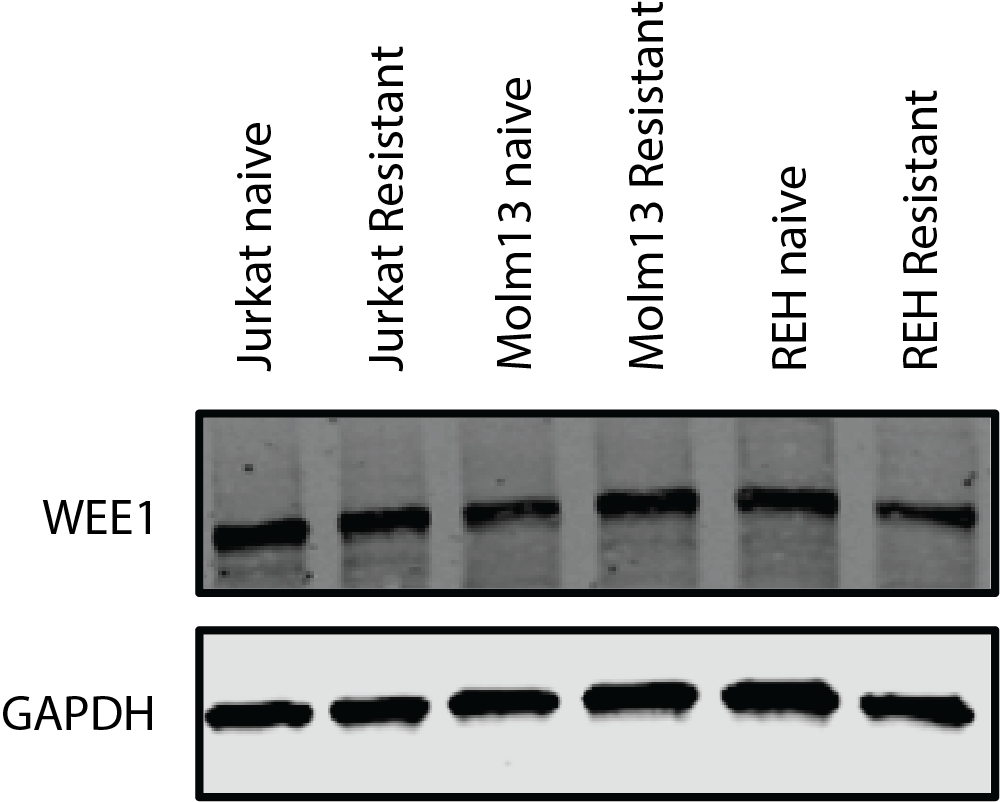


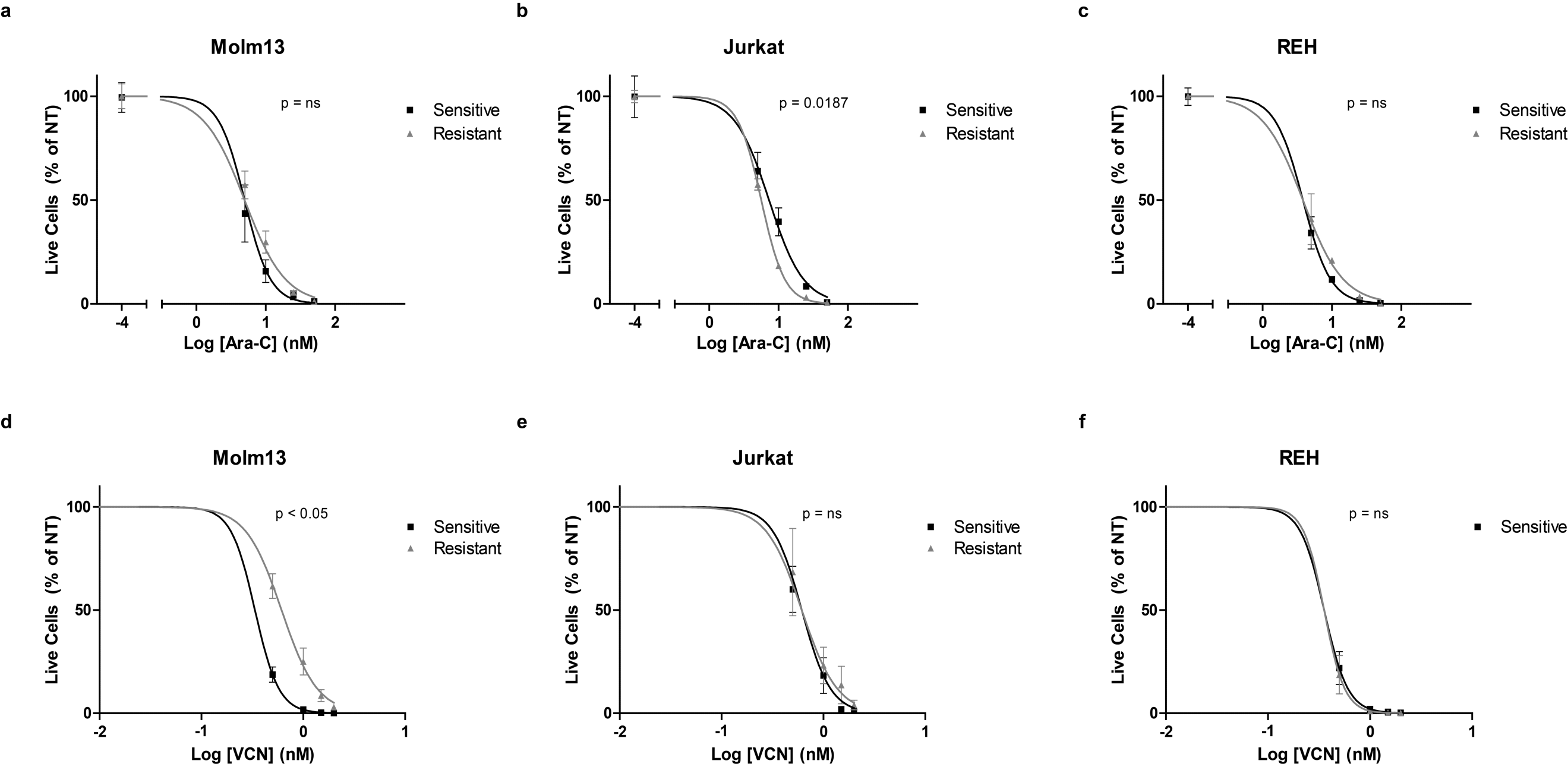
**Supplementary Figure 2. AZD1775-resistant cells are not broadly resistant to DNA damaging agents.** (**a-c**) AZD1775-sensitive and -resistant Molm13 (**a**), Jurkat (**b**), and REH (**c**) cells were treated with 5-50 nM Ara-C for 72 hr. Viable cell counts normalized to cells receiving no treatment (NT) are shown. Results are shown as mean ± SEM from three independent experiments. (**d-f**) AZD1775-sensitive and -resistant Molm13 (**d**), Jurkat (**e**), and REH (**f**) cells were treated with 0.5-2 nM vincristine (VCN) for 72 hr. Viable cell counts normalized to cells receiving no treatment (NT) are shown. Results are shown as mean ± SEM from three independent experiments.


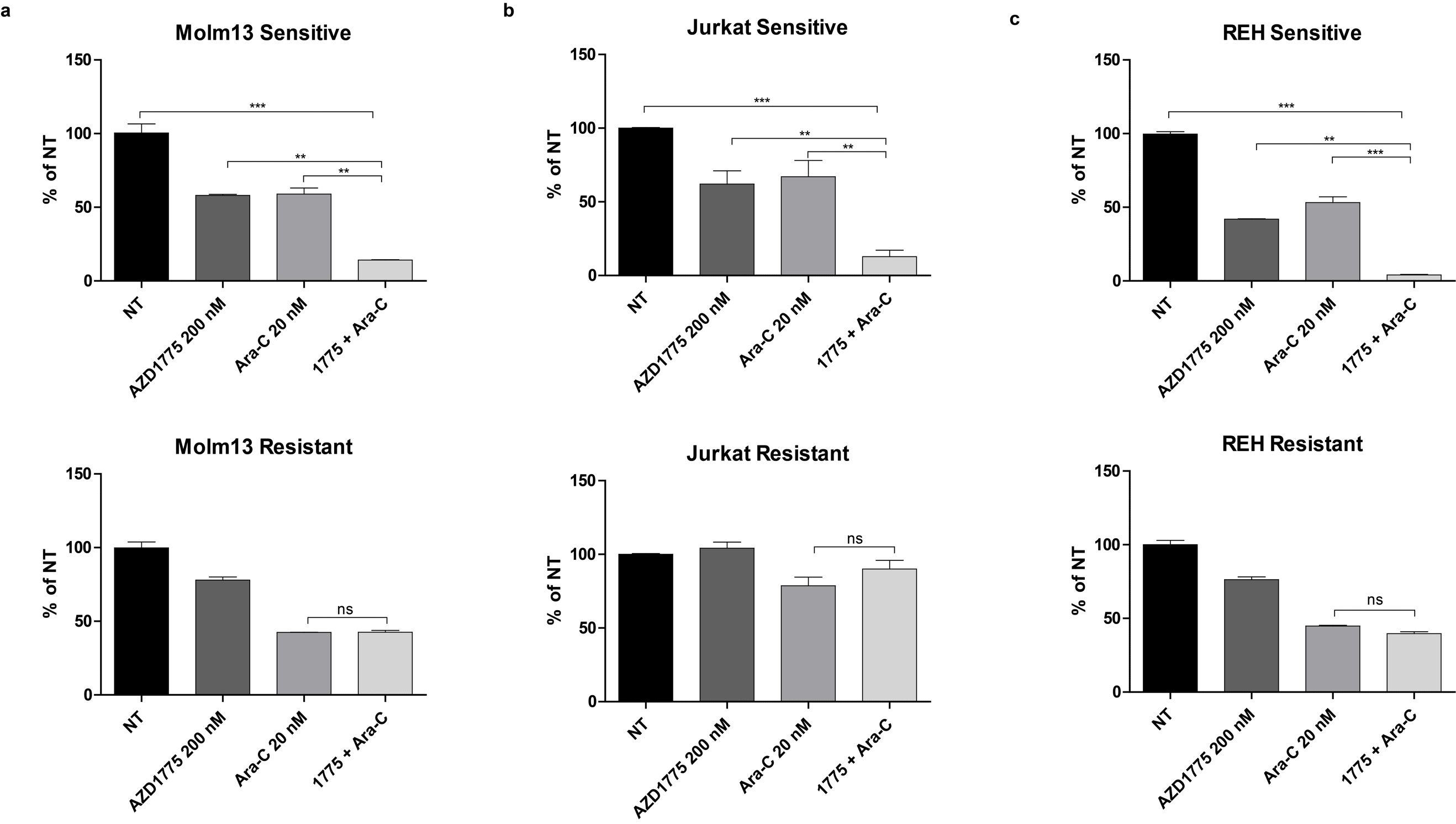
**Supplementary Figure 3. AZD1775-resistant cell lines are resistant to AZD1775 combined with Ara-C.**  AZD1775-sensitive and -resistant Molm13 (**a**), Jurkat (**b**), and REH (**c**) cells were treated with AZD1775 (200 nM) and/or Ara-C (20 nM) for 72 hr. Viable cell counts are normalized to cells receiving no treatment (NT). Results are displayed as mean ± SEM from a minimum of two independent experiments. **, P < 0.01. ***, P < 0.001.


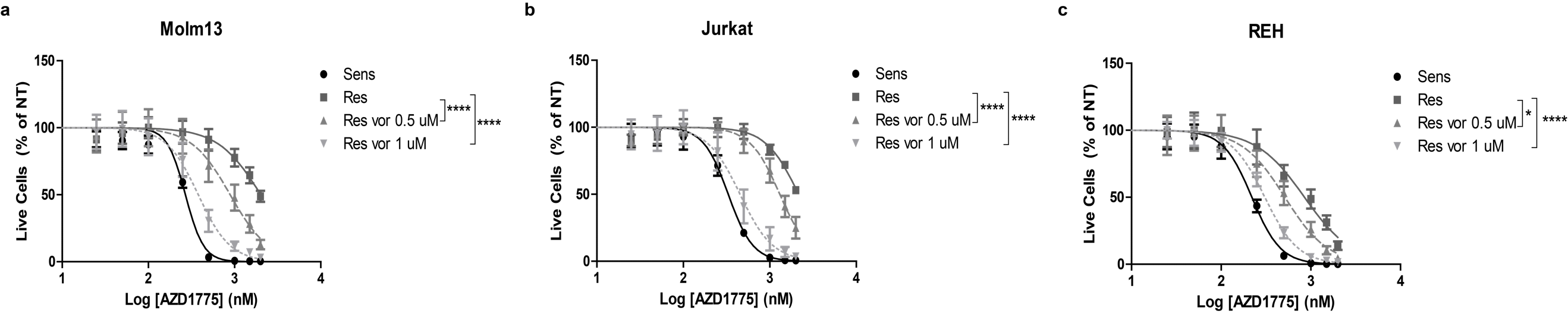
**Supplementary Figure 4. Vorinostat sensitizes resistant cells to AZD1775.** (**a-c**) AZD1775-sensitive and -resistant Molm13 (**a**), Jurkat (**b**), and REH (**c**) cells were treated with the indicated concentrations of vorinostat and/or 25-2000 nM AZD1775 for 72 hr. Viable cell counts normalized to cells receiving no AZD1775 treatment (NT) are shown. Results are shown as mean ± SEM from a minimum of three independent experiments. *, P < 0.05. ****, P < 0.0001.


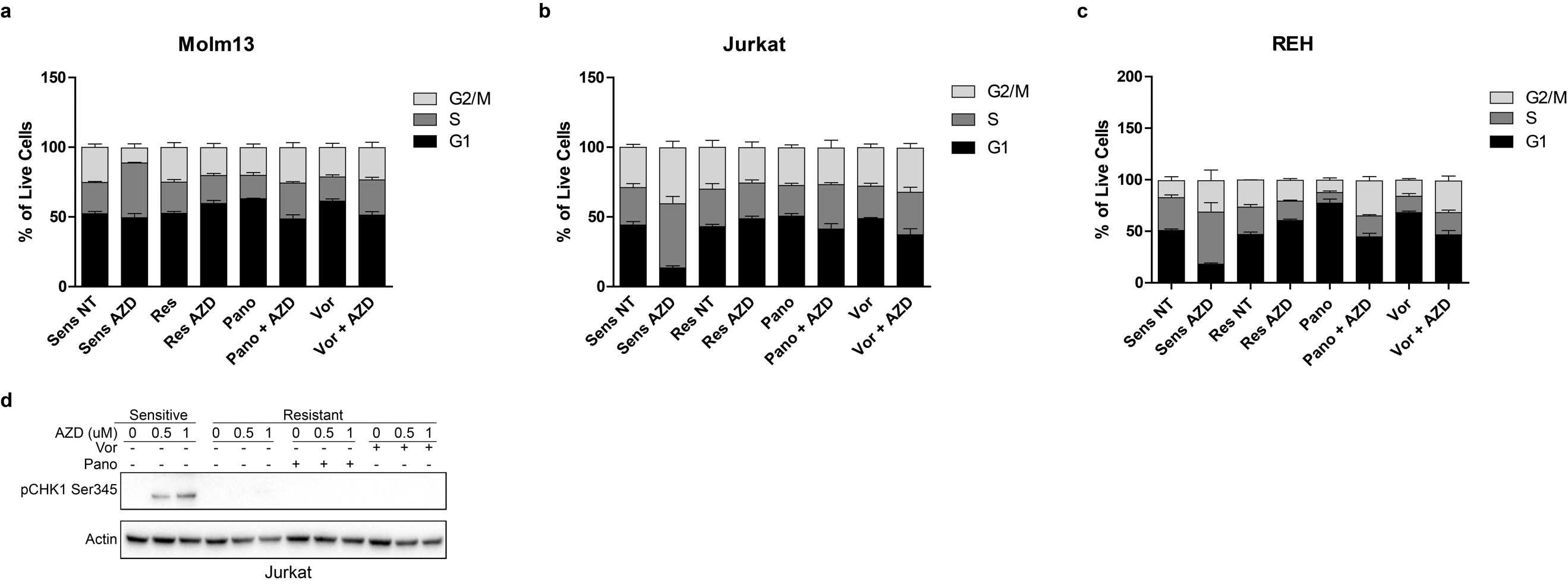
**Supplementary Figure 5. Cell cycle analysis and CHK1 phosphorylation in AZD1775-sensitive and resistant cells treated with HDAC inhibitors and/or AZD1775.** (**a-c**) AZD1775-sensitive and -resistant Molm13 (**a**), Jurkat (**b**), and REH (**c**), cells were treated with panobinostat (10 nM), vorinostat (1 μM), and/or AZD1775 (1 μM) for 24 hr then fixed and stained with propidium iodide (Guava Cell Cycle reagent, Millipore). Results are shown as mean ± SEM from three independent experiments. (**d**) AZD1775-sensitive and -resistant Jurkat cells were treated with panobinostat (10 nM), vorinostat (1 uM), and/or AZD1775 (0.5 μM or 1 μM) for 24 hr after which protein lysates were subjected to western blotting with antibodies specific to pCHK1 serine 345 and actin (Cell Signaling Technology).


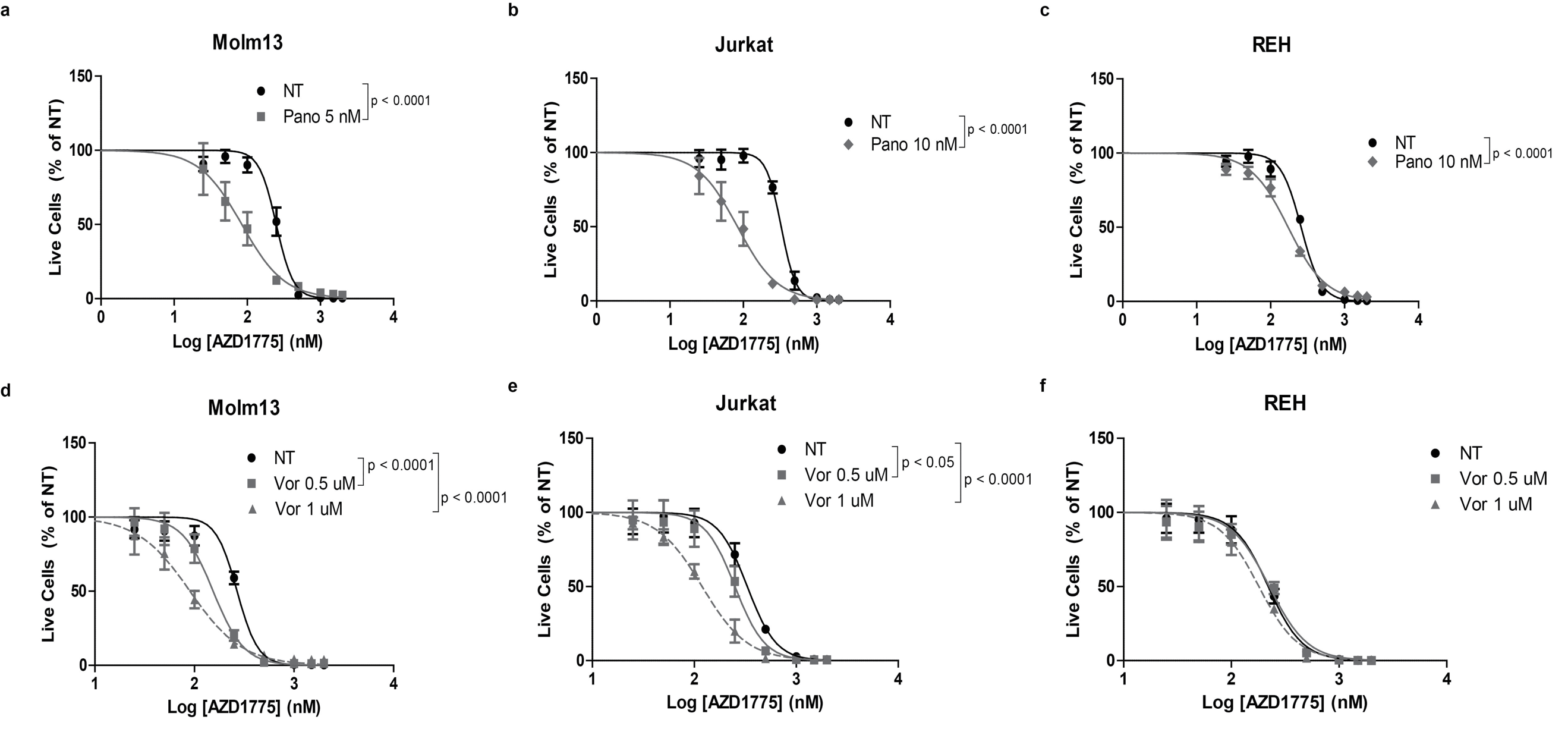
**Supplementary Figure 6. HDAC inhibition enhances the anti-proliferative effects of AZD1775 in cells without acquired AZD1775 resistance.** (**a-c**) AZD1775-sensitive Molm13 (**a**), Jurkat (**b**), and REH (**c**) cells were treated with the indicated concentrations of panobinostat and/or 25-2000 nM AZD1775 for 72 hr. Viable cell counts are normalized to cells receiving no AZD1775 treatment (NT). Results are shown as mean ± SEM from a minimum of 3 independent experiments. (**d-f**) AZD1775-sensitive Molm13 (**d**), Jurkat (**e**), and REH (**f**) were treated with the indicated concentrations of vorinostat and/or 25-2000 nM AZD1775 for 72 hr. Viable cell counts are normalized to cells receiving no AZD1775 treatment (NT). Results are shown as mean ± SEM from a minimum of 3 independent experiments.

**Supplementary Figure 7. AZD1775-resistant Jurkat cells have elevated c-MYC expression which is abrogated with panobinostat treatment. A.** Ingenuity Pathways Analysis network map of gene fold change values of AZD1775-resistant Jurkat cells treated with AZD1775 (1000 nM) compared with AZD1775-sensitive Jurkat cells treated with AZD1775 (1000 nM). Genes in red have increased expression in resistant cells while those in green have decreased expression in resistant cells compared with sensitive cells. **B.** Ingenuity Pathways Analysis network map of gene fold change values of AZD1775-resistant Jurkat cells treated with panobinostat (10 nM) and AZD1775 (1000 nM) compared resistant cells treated with AZD1775 (1000 nM) alone. Genes displayed in red have increased expression while those displayed in green have decreased expression in resistant cells treated with panobinstat and AZD1775 compared with cells treated with AZD1775 alone.

**
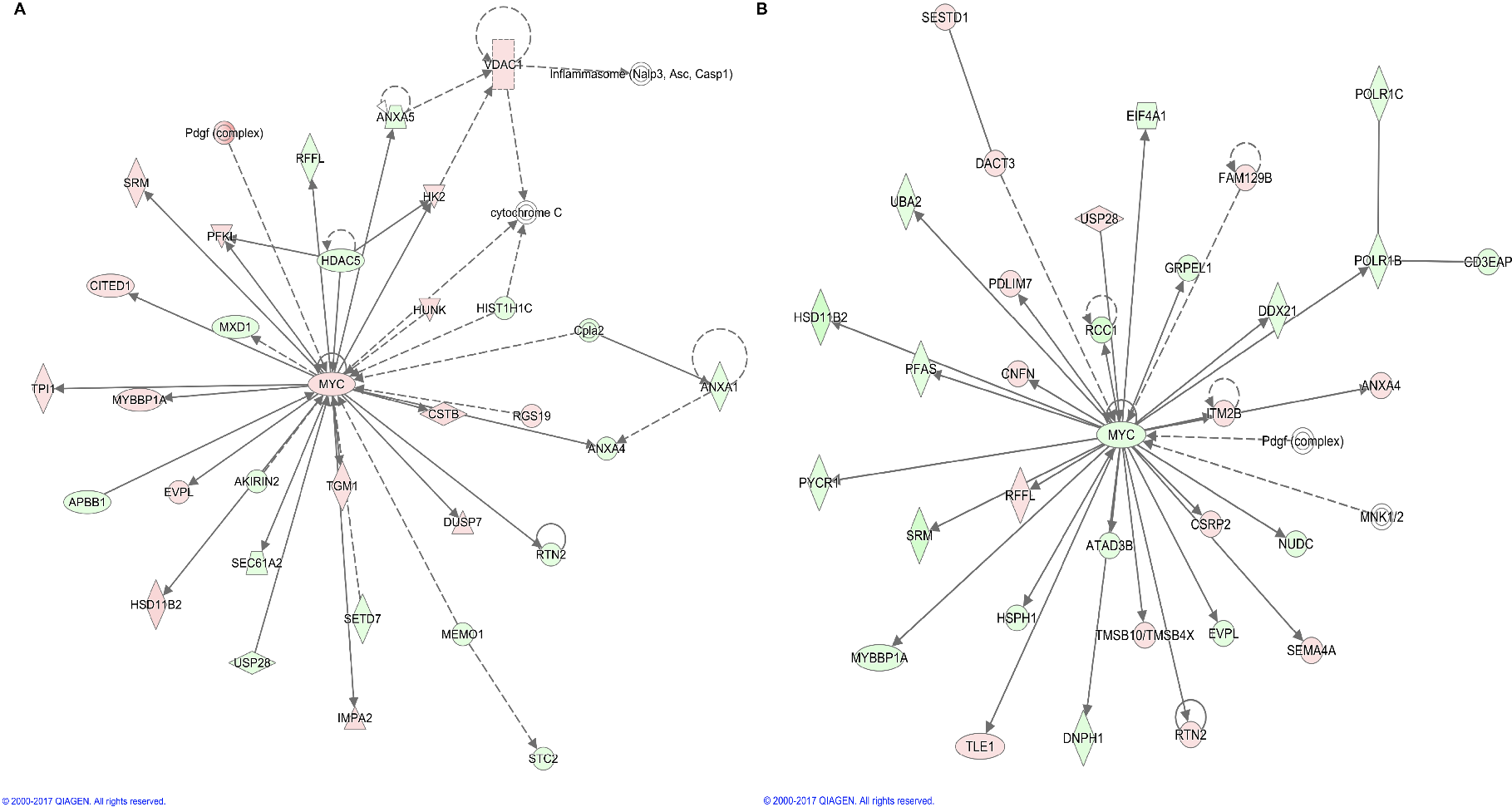
**
